# The Life of a Travelling Physician, from His First Introduction to Practice; Including Twenty Years' Wanderings through the Greater Part of Europe

**Published:** 1843-07-01

**Authors:** 


THE LIFE OF A TRAVELLING PHYSICIAN, FROM HIS FIRST INTRODUCTION TO PRACTICE; INCLUDING TWENTY YEARS' WANDERINGS THROUGH THE GREATER PART OF EUROPE. In three Volumes 8vo. Longman and Co. 1843.

FROM internal evidence in these volumes we think and hope that they do not embrace more than half of the "travelling physician's life"—though they may do that of "the *travelling life*" of the physician. The lively author arrived in this metropolis fresh from the schools of "Auld Reekie" in 1819, bringing with him a whole portfolio of introductory letters, not only to people in London, but residents in other countries. His health was bad in Scotland, and a warm climate was his object. But the voyage (not then by steam) completely restored him, and he was fit for anything. His reception by various medical men was very ludicrous—varying from the politeness, and even kindness of Sir Astley Cooper, down to the surly, unmannerly, and insulting audience of an overgrown aristocratic apothecary—a genus now pretty nearly extinct. After many ineffectual attempts to get into a dispensary, as the road to practice, our author obtained an appointment as domestic physician to a nobleman far advanced in consumption, and commencing his journey to a southern clime. Then did our author enter upon his "travelling life." These kinds of appointments present more attractions than solid advantages to the young physician. We have rarely seen them turn out well in the long run. They are not equal, in any point of view, to the Army, Navy, or East India Service. They are shewy, toil-less, well-fed kinds of life; but, excepting for three or four years, between graduation at the schools and settlement in private practice, they are worse than useless, as far as regards success and reputation in the private walks of the profession. "Mais chacun a son gout."

The journey from London to PAU, for that was the final destination of the noble invalid, does not present anything that we can extract into a medical journal. It is cleverly written—as are the whole of the volumes—but not very profound; and scattering but very few particles of professional information or reflection in the general stream of description, anecdote and travelling gossip.

As might have been anticipated, the noble patient sank at Pau, and left his bones at Orthez. Our author makes very few remarks on the climate of this favourite locality, and still fewer on the mineral waters of the Pyrennees. The following passage is all that we can extract.

"With respect to the climate I can say but little in its favour. The cold was often very severe; and in the month of January the thermometer (Reaumur's) during three or four days, marked 20 degrees of frost. The air was at all times sharp; and the mountains invited the clouds to break upon them when their contents were about the freezing point, so that there was much cold rain in the Autumn. With the exception of these few days of severe frost the Winter much resembled such as I had remembered it in the middle counties of England. There was, certainly, as far as concerned temperature alone, nothing which should induce an invalid to leave an English climate, with the hope of finding a better at Pau. This, however, was a most extraordinary season. Some credit might be given to the assertion, as the cold was generally severe in the south of Europe. At Nice, the orange trees perished; but, upon the whole, we had not much to complain of. The Autumn was long, and there were many fine hours, which could be turned to good account, and then there was the *petit été de St. Martin,* which the French boast of in these latitudes, though sometimes it skips over Martinmas, and ushers in the Spring." 53.

The last rites performed to the remains of the nobleman, our author returned to London, and, by the advice of a friend in Thavies Inn, made a trial for a Dispensary. The adventures during the canvas are humorous and well told; but, in those days, a *new* batch of subscribers could turn the scale, and our author's purse not being so heavy as that of his competitor, he lost the day.

From domestic MEDICO to a Lord, the travelling physician became the inmate of a Prince's establishment in Paris, at a salary of £500. per annum, and the engagement for five years. We must pass over the whole of this period, for the professional narrative is almost nul; though the French capital might have afforded him a vast fund of medical and surgical information, had his propensities lain in that direction. The author became acquainted with Gall and Spurzheim, and plainly says that phrenology is the baseless fabric of a vision. In this we do not agree with him, but let that pass.

From Paris our author travelled with Prince —— towards Poland, giving slight sketches of the principal localities through which he passed. At

Carlsbad our author gives way to the usual exaggeration of the Sprudel leaping seven or eight feet high, and covering the valley with mineral vapour. His residence in Cracow enables him to give an animated description of the wretched state of the Jews in that Republic of Liberty. His remarks on the Poles themselves would lead us not to deplore their loss of national freedom and independence, of which they were unworthy. A Polish Nobleman, when he killed one of his serfs or peasants, was punished by the expenses of burying the murdered man—that was all! Our author's description of a Russo-Polish dinner on their route from Cracow to Odessa, is a real gastronomic curiosity, well deserving the study of all those who are investigating the physiology of digestion. It was at the castle of an octogenarian lady, who had long figured in the court circles of Catherine of Russia.

"Behind each person stood a servant, not dressed in the most splendid livery. The dinner commenced by slices of cold ham handed round in a dish; then a cold *pâté* of the livers of geese; then a salad consisting of craw fish garnished with slices of beet root, and, lastly, some thin slices of Parmesan cheese.

"Being myself fond of cold meats, I congratulated myself upon having made a good dinner, though I could have devoured more with pleasure; but as I saw the other guests help themselves but sparingly I could but follow their example. I was about to ask for a third slice of bread, having consumed the two portions of white and brown which were placed before me, when I opened the eyes of astonishment upon the entry of the soup. Not knowing how to act, I watched the operations of the Countess, thinking that I could not do wrong if I followed her example. I despatched a plate full of craw fish soup, than which I never tasted any thing more exquisite, and, seeing the hostess qualify it with a glass of wine, I filled my glass from a bottle near me; the doctor's place being, as I have before observed, at the end of the table. Whether she perceived any wryness in my face as I gulped down the sour wine, I know not, but she ordered the man behind her chair to put beer and kvass upon the board, and immediately a bottle of each was placed before me. I partook of both during the repast, but they were not to my taste. I now found a large sirloin of beef at my left shoulder. The countess had already helped herself very plentifully, but, after having tasted a mouthful or two, she sent her plate away, which she did with two thirds of the dishes. I found that a favourite servant enjoyed the privilege of eating off her mistress's plate, who was now employed in groping with her fork in a black earthenware jug, from the top of which a bladder had been partially removed, to pick out some stewed kidneys, which she consumed with a peculiar gusto. This dish was not handed round. Some buckwheat, boiled, and served up with cold butter in a saucer, followed the beef. I took the liberty of allowing this dish to pass, having indeed dined before the arrival of the soup: as I saw in what way the hostess treated her platefuls, I was easy upon this score. The next temptation presented itself in the shape of stewed carp, of which I partook, but they had the real muddy taste of the species; they were well dressed, and seemed to be approved. Had the wine been better, it might have stimulated my stomach to a little longer warfare; as it was, I was quite *hors du combat,* and saw with pleasure what I supposed to be the last dish, in some chickens stuffed with parsley. I had often heard that eating and drinking to excess were very hard labour, and I seemed to be proving the truth of the adage; the chickens being handed to me, I summoned up courage and took a wing to play with; and on my plate being removed, I found a plum pudding at my elbow. Not venturing to attack this dish (the *mehlspeise* of the Germans), another was presented, consisting of fine asparagus covered with a sweet sauce. I had no alternative but to die of an apoplexy, or cease to eat altogether. I preferred the latter. I had now only to gaze and wonder at the capacity of the guests' stomachs, most of whom partook of every dish which was presented to them, and many asked me why I did not eat. The asparagus was succeeded by an immense joint of roast veal, served with salad, and the repast was terminated by a pile of cold craw fish, which were picked and eaten as a kind of *passe tempe*. Little conversation, or only monosyllabic dialogues enlivened the meal: all seemed anxious to lay in a stock of the *vis vitæ* only." Vol. 2, p. 43.

How would Abernethy have stared had he been present at the above dinner! How would Jephson have stamped, and ordered the dishes off the table! It is scarcely credible, were it not authenticated by daily observation, that the human stomach can receive—to digest is out of the question—such a monstrous mass of heterogeneous materials at one meal!

On their way to Odessa they encountered a cloud of locusts that darkened the air, and destroyed every particle of green vegetable on which they alighted. At Odessa, where the Imperial Court resided at the time, our author appears to have picked up a tolerable harvest in the fee trade; but as the land of locusts did not present a good field for fattening a physician, our travelling physician counted his rubles, and started for St. Petersburg, in company with one of his own countrymen, who was also an adventurer in life, but not of the medical profession. The journey is described amusingly, like all other parts of the work, but offers nothing for medical remark.

Our author had obtained permission from the Emperor Alexander at Odessa to practise his profession throughout Russia, withont submitting to an examination at St. Petersburg, and having been graciously received by the Minister of the Interior, and the President of the Medico-Chirurgical Academy—his name was registered among the free practitioners—and now only wanted patients to make him a metropolitan instead of a "travelling physician." He soon got a patient—in the person of his female servant—who had a diarrhœa. He does not say whether or not he cured her. He then waited on all the grandees and others to whom he had recommendations.

"Some of the parties encouraged me to hope for success, as most of my countrymen had found it in Russia. Others made me polite bows, and received me standing. Some would not receive me at all." Vol. 2, p. 177.

After a considerable interval he was summoned to attend a Russian Princess—but princes and princesses are as plentiful in Russia as in Naples or Sicily—that is, like blackberries. In Russia a prince drove a hackney-coach for his bread. After the princess he had not a patient for six months, and began to look very blue on the occasion, especially as the thermometer was a huge way below Zero. At length, on the 1st January, 1829, the chief English physician at St. Petersburg (we suppose Sir A. Crichton) resigned his practice to our author. "Now it was that the professional scale turned in my favour—now it was that I was to receive succour from an unexpected source—now it was that FATE triumphed over plans and systems." "During the remainder of my sojourn in Russia, I may briefly state that I have practised upon a moderately extensive scale." "As these extracts are *not* from professional life—we may say no more about the matter."

From the concluding passage, it is probable that our author may have in reserve his "professional memoirs," which he did not like to mix up with his "travelling life." We see no reason why a physician should not write as good a book of travels as any other person, provided he has had a liberal education, independent of his medical studies. On the contrary, we think the medical tourist has advantages peculiar to his profession, which may and have been turned to good account, both in travelling and in writing travels. We need only instance two living authors—Holland and Madden, as sufficient for our purpose. The present work, if we may be allowed to give an opinion, is written in a lively, often facetious style—sometimes, but not always, interspersed with deep reflection and acute observation. Political opinions, apparently from the mouths of other personages, are, we suspect, his own, and these will not find favour in every reviewer's sight. The author may, therefore, expect a few hornets about his ears, more troublesome than even the locusts who surrounded him in the Ukraine! We should like to see a key to all the blanks in these volumes. Some of the personages whose portraits are here drawn, we think we have seen. There is one which *appears* somewhat caricatured, and in it we imagine that we recognize Sir James Wylie, Bart. Physician to the late Emperor Alexander—but we may be wrong. We return our best thanks to the unknown, or at least anonymous author, for the entertainment and information we have derived from his amusing life, which we hope will be prolonged so that he may be able, twenty years hence, to give us three more volumes of professional travels through modern Babylon.

